# Advantage of the F2:A1:B- IncF Pandemic Plasmid over IncC Plasmids in *In Vitro* Acquisition and Evolution of *bla*_CTX-M_ Gene-Bearing Plasmids in Escherichia coli

**DOI:** 10.1128/AAC.01130-19

**Published:** 2019-09-23

**Authors:** Anne-Claire Mahérault, Harry Kemble, Mélanie Magnan, Benoit Gachet, David Roche, Hervé Le Nagard, Olivier Tenaillon, Erick Denamur, Catherine Branger, Luce Landraud

**Affiliations:** a Université de Paris, IAME, UMR 1137, INSERM, Paris, France; b AP-HP, Laboratoire de Microbiologie Hygiène, Hôpital Louis Mourier, Colombes, France; c Laboratoire d’Analyse Bioinformatique en Génomique et Métabolisme, Institut de Génomique, Commissariat à l’Energie Atomique, Génoscope, CNRS-UMR 8030, Evry, France; d AP-HP, Laboratoire de Génétique Moléculaire, Hôpital Bichat, Paris, France

**Keywords:** CTX-M-15, *Escherichia coli* ST131, IncC plasmid, IncF plasmid, experimental evolution, extended-spectrum beta-lactamase, fitness cost

## Abstract

Despite a fitness cost imposed on bacterial hosts, large conjugative plasmids play a key role in the diffusion of resistance determinants, such as CTX-M extended-spectrum β-lactamases. Among the large conjugative plasmids, IncF plasmids are the most predominant group, and an F2:A1:B- IncF-type plasmid encoding a CTX-M-15 variant was recently described as being strongly associated with the emerging worldwide Escherichia coli sequence type 131 (ST131)-O25b:H4 *H*30Rx/C2 sublineage. In this context, we investigated the fitness cost of narrow-range F-type plasmids, including the F2:A1:B- IncF-type CTX-M-15 plasmid, and of broad-range C-type plasmids in the K-12-like J53-2 E. coli strain. Although all plasmids imposed a significant fitness cost to the bacterial host immediately after conjugation, we show, using an experimental-evolution approach, that a negative impact on the fitness of the host strain was maintained throughout 1,120 generations with the IncC-IncR plasmid, regardless of the presence or absence of cefotaxime, in contrast to the F2:A1:B- IncF plasmid, whose cost was alleviated. Many chromosomal and plasmid rearrangements were detected after conjugation in transconjugants carrying the IncC plasmids but not in transconjugants carrying the F2:A1:B- IncF plasmid, except for insertion sequence (IS) mobilization from the *fliM* gene leading to the restoration of motility of the recipient strains. Only a few mutations occurred on the chromosome of each transconjugant throughout the experimental-evolution assay. Our findings indicate that the F2:A1:B- IncF CTX-M-15 plasmid is well adapted to the E. coli strain studied, contrary to the IncC-IncR CTX-M-15 plasmid, and that such plasmid-host adaptation could participate in the evolutionary success of the CTX-M-15-producing pandemic E. coli ST131-O25b:H4 lineage.

## Abstract (precis)

Despite a fitness cost imposed on bacterial hosts, large conjugative plasmids play a key role in the diffusion of resistance determinants, such as CTX-M extended-spectrum β-lactamases. Among the large conjugative plasmids, IncF plasmids are the most predominant group, and an F2:A1:B- IncF-type plasmid encoding a CTX-M-15 variant was recently described as being strongly associated with the emerging worldwide Escherichia coli sequence type 131 (ST131)-O25b:H4 *H*30Rx/C2 sublineage.

## INTRODUCTION

Dissemination of multiresistant clinical bacterial strains is a major concern worldwide (1). In particular, the increase in resistance to expanded-spectrum cephalosporins (ESCs), mainly due to extended-spectrum β-lactamases (ESBLs), constitutes a challenge to treating *Enterobacteriaceae* infections. Since the 2000s, a radical shift in the distribution of ESBLs has occurred. TEM and SHV ESBL enzymes, predominant in the 1980s and 1990s, have become outnumbered by the CTX-M enzymes (2, 3). Many recent epidemiological studies throughout the world now describe the CTX-M-15 and CTX-M-14 variants as the most widely dominant among CTX-M β-lactamases (4, 5). Among the ESBL-producing *Enterobacteriaceae*, Escherichia coli has increased exponentially over the past 20 years (6). Moreover, ESBL-producing E. coli strains are an emerging problem in community-acquired infections and have also increasingly been detected in food-producing, companion, and wildlife animals as well as in the environment (7, 8). Several major pandemic multidrug-resistant clones of E. coli have emerged and contributed to the dissemination of the ESBLs (9–11). Among them, an E. coli clone of phylogenetic group B2, sequence type 131 (ST131), and serotype O25b:H4 is strongly associated with the global spread of the *bla*_CTX-M-15_ gene (12–15).

Large conjugative plasmids are associated with multiresistance in *Enterobacteriaceae* (16–18). Through horizontal transfer, they contribute to the spread of resistance traits. Three incompatibility (Inc) groups of plasmids, the narrow-host-range IncF and IncI1 plasmids and the broad-host-range IncC plasmid, formerly IncA/C_2_, are prevalent and associated with ESBL-producing *Enterobacteriaceae* strains (16, 18–21). However, in E. coli strains that produce CTX-M ESBL, the *bla*_CTX-M-15_ genes are located mainly on plasmids belonging to the IncF group (4, 11, 17, 18), whereas the *bla*_CTX-M-14_ genes are carried on a variety of plasmid types, including IncF ones (4, 16, 18, 20). Although IncF plasmids carrying the *bla*_CTX-M-15_ gene are not exclusive to the ST131 clone, since they were identified in other E. coli STs (ST405, ST354, ST28, and ST695) (10, 11, 16, 19), an association has recently been observed between the pandemic multiresistant E. coli O25b:H4 ST131 *H*30Rx/C2 sublineage and the IncF plasmid of replicon sequence type (RST) F2:A1:B- (22).

Although the carriage of conjugative resistance plasmids initially confers a fitness cost (23, 24), unexpected stabilization and spread of the conferred resistance have been observed in bacterial populations, even in the absence of antibiotic selection, with the pandemic success of some resistant clones. A complex balance between specific plasmid characteristics (cost, conjugative efficiency, and genes encoding stabilization functions), positive epistatic interactions between coresident plasmids within the same bacterial host, and various evolutionary mechanisms of plasmid-host associations could explain the long-term persistence of multidrug-resistant plasmids in bacterial populations (25–28). Experimental-evolution studies have been performed in various bacterial models to understand how the adaptation of an antibiotic resistance plasmid to a new bacterial host could lead to its persistence (24, 29, 30). Recently, analysis of these mechanisms at the genomic level has contributed to a better understanding of these adaptive processes (31–33). It has been shown that plasmid-host interactions leading to genetic changes in both plasmid and chromosome can contribute to a lineage’s evolutionary success (34–36). Nevertheless, the phenomenon of plasmid-host stabilization does not depend on a universal mechanism but probably varies according to the plasmid-host combination and plasmid background (37, 38). Hypotheses such as selective pressure exerted by the use of antimicrobial drugs and/or emergence of competitive E. coli clones carrying well-adapted F-type plasmids have been proposed to explain the global spread of E. coli strains associated with IncF plasmids carrying the *bla*_CTX-M-15_ gene (8, 15, 22, 39). However, to the best of our knowledge, no experimental-evolution study has yet been performed to analyze the adaptive process of these plasmids.

In this context, using an experimental-evolution approach coupled with whole-genome sequencing (WGS), we compared the fitness costs of narrow-range F-type plasmids, including the IncF CTX-M-15 plasmid of RST F2:A1:B-, and of broad-range C-type plasmids in the K-12-like J53-2 E. coli strain.

## RESULTS

### Cost of multidrug resistance IncF and IncC plasmids in the J53-2 E. coli recipient strain.

To evaluate the plasmid cost to bacterial fitness, we used three narrow-host-range IncF plasmids (pRCS59 and pRCS102 carrying a *bla*_CTX-M-15_ gene and pRCS65 carrying a *bla*_CTX-M-14_ gene) and two broad-host-range IncC plasmids (pRCS30 carrying a *bla*_CTX-M-14_ gene and pRCS46 carrying a *bla*_CTX-M-15_ gene), selected from a previous collection of well-characterized ESBL-encoding plasmids originating from human clinical E. coli strains and recently sequenced (40) (see Fig. S1 in the supplemental material). The *bla*_CTX-M-15_ IncF plasmids pRCS59 and pRCS102 are of RST F2:A1:B- (41) and have an overall structure similar to that of the archetypal F2:A1:B- *bla*_CTX-M-15_ plasmid described by Johnson et al. as being associated with the ST131 *H*30Rx/C2 fluoroquinolone-resistant sublineage of E. coli (22). The *bla*_CTX-M-15_ pRCS46 IncC plasmid is of plasmid multilocus sequence type 3 (pST3) (https://pubmlst.org/plasmid/) (42, 43) and has the particularity of being a composite plasmid that has integrated an IncR plasmid within a large sequence inserted at an ARI-A integration spot (IncC-IncR plasmid) (21, 44, 45). In addition to the *bla*_CTX-M_ genes, all the plasmids carry multiple other resistance genes (for additional information, see Materials and Methods and Fig. S1). The sizes of the plasmids vary from 121 kb to 130 kb for the IncF plasmids and are 157 kb and 216 kb for the IncC plasmids pRCS30 and pRCS46, respectively (40).

Multiple independent transfers of the five plasmids into E. coli J53-2 plasmid-free strains were conducted to obtain multiple independent transconjugants (TRs): TR59-IncF (J53-2/pRCS59), TR102-IncF (J53-2/pRCS102), TR65-IncF (J53-2/pRCS65), TR30-IncC (J53-2/pRCS30), and TR46-IncC-IncR (J53-2/pRCS46). Transfer of the IncF plasmids was poorly efficient (30% to 40%) compared to the IncC plasmids (100%).

**FIG 1:**
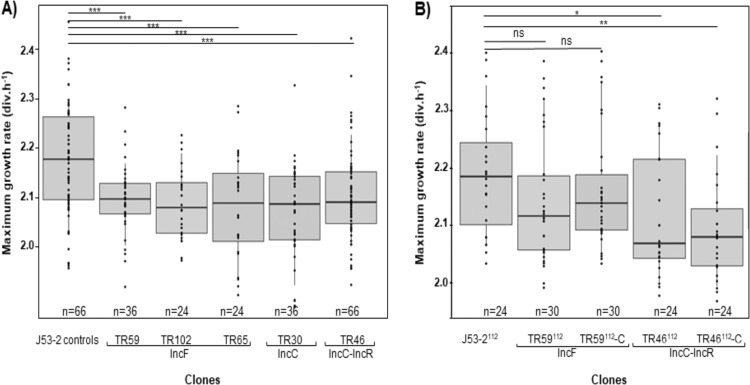
Box plot representation of the maximum growth rate (MGR) of each pool lineage. (A) MGRs of 11 J53-2 controls (J53-2^rec^ and J53-2^inc^) and 41 transconjugants: 6 TR59 (pRCS59, IncF, F2:A1:B-), 4 TR102 (pRCS102, IncF, F2:A1:B-), 4 TR65 (pRCS65, IncF, F2:A-:B-), 6 TR30 (pRCS30, IncC, pST3), and 11 TR46 (pRCS46, IncC-IncR, pST3). (B) MGRs of evolved J53-2, TR59, and TR46 at day 112 (indicated in superscript), with or without cefotaxime (-C). For each box, the central mark indicates the median, and the bottom and top edges of the box indicate the 25th and 75th percentiles, respectively. Each point corresponds to one measurement per clone, with *n* corresponding to the number of measurements, which were repeated six times per transconjugant and J53-2 control. Asterisks indicate significant differences (ns, not significant; *, *P* < 0.05; **, *P* < 0.01; ***, *P* < 0.001).

Immediately after conjugation, we measured the maximum growth rates (MGRs) of 31 of the 40 transconjugants obtained (6 TR59-IncF, 4 TR102-IncF, 4 TR65-IncF, 6/10 TR30-IncC, and 11/16 TR46-IncC-IncR). The MGRs were compared to the MGRs of 6 of 21 freshly isolated E. coli J53-2 plasmid-free strains (J53-2^rec^) and 5 of 20 E. coli J53-2 plasmid-free strains having been incubated concomitantly with the transconjugants (J53-2^inc^) (for details, see Materials and Methods) (Fig. 1A). First, we compared the MGRs of the strains in each group of transconjugants with E. coli J53-2 strains. As no significant fitness cost variation was detected, we performed further statistical analyses after pooling the MGRs of the strains of each group. E. coli J53-2 (J53-2^rec^ and J53-2^inc^) strains showed an MGR of 2.18 divisions (div) h^−1^, significantly higher than those of the TR59-IncF (2.09 div h^−1^), TR102-IncF (2.08 div h^−1^), TR65-IncF (2.09 div h^−1^), TR30-IncC (2.08 div h^−1^), and TR46-IncC-IncR (2.09 div h^−1^) transconjugants (*P* < 0.001) (Fig. 1A).

### Whole-genome analysis of transconjugants.

To identify mutations and rearrangements that may occur after conjugation, we sequenced all 40 transconjugants obtained from the independent conjugation assays (6 TR59-IncF, 4 TR102-IncF, 4 TR65-IncF, 10 TR30-IncC, and 16 TR46-IncC-IncR) and J53-2 controls (21 J53-2^rec^ strains and 20 J53-2^inc^ strains). For genomic comparison, we used the open-source Breseq pipeline (46) and the Polymorphism Analyses in Light of Massive DNA Sequencing (PALOMA) approach, provided by the MaGe platform (47).

In a first step, using the E. coli K-12 MG1655 strain as a reference chromosomal genome, we excluded all mutations or rearrangements common to all J53-2 plasmid-free genomes and all transconjugant genomes. In a second step, we determined genetic differences occurring between the J53-2 genomes and the transconjugant genomes. Point mutations detected both in J53-2 genomes and in transconjugant genomes, or only in J53-2 genomes, were excluded from further analyses (four intergenic point mutations and two nonsynonymous point mutations in the *pykF* and *rpoC* genes) (Fig. S2).

**FIG 2:**
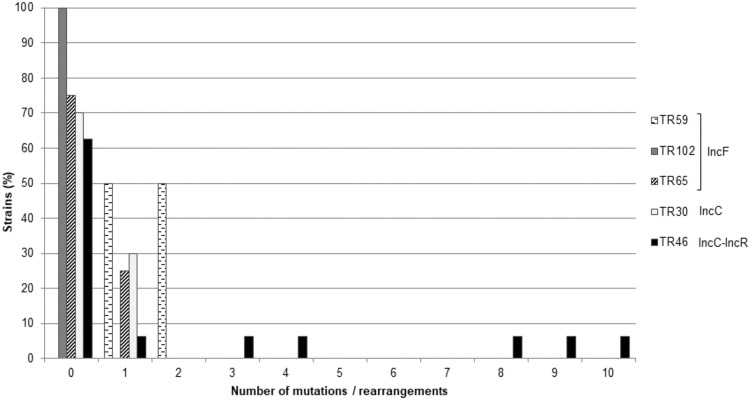
Distribution of strains (expressed as a percentage) of each transconjugant group (TR59, *n* = 6; TR102, *n* = 4; TR65, *n* = 4; TR30, *n* = 10; TR46, *n* = 16) related to the number of mutations and rearrangements detected in the chromosome (0 to 10).

In chromosomal DNA, we detected 23 different events between J53-2 strains and transconjugants (Fig. S2 and Table S1). In all TR59-IncF transconjugants, the IS*150* insertion interrupting the *fliM* gene that is present on the J53-2 genome was mobilized. In addition, three other events were respectively found in three TR59-IncF transconjugants: an IS*5* insertion in an intergenic region and two point mutations (one intergenic point mutation and one synonymous point mutation in the *metB* gene). No difference was detected between J53-2 strains and TR102-IncF transconjugants. One chromosomal point mutation was detected between J53-2 strains and one TR65-IncF transconjugant (a synonymous point mutation in the *yagF* gene). Three differences were detected between J53-2 strains and TR30-IncC transconjugants: two nonsynonymous point mutations (*ydfU* and *hslR* genes), each in a single transconjugant, and one rearrangement in a third transconjugant (an IS*2* insertion in the *rhsA* gene). Eighteen differences were detected between J53-2 strains and TR46-IncC-IncR transconjugants: 12 point mutations (2 intergenic, 9 nonsynonymous, and 1 synonymous point mutations) and 6 rearrangements (1 CPZ-55 prophage insertion and 5 insertion sequence [IS] mobilizations, including the IS*150* mobilization from the *fliM* gene). Of the nine nonsynonymous point mutations in the *ybdK*, *bioF*, *acnA*, *ydfU*, *fadH*, *rpoN*, *nirB*, *damX*, and *yjiN* genes, four were predicted to be deleterious according to four distinct metrics (SIFT, Polymorphism-Phenotyping V2 [Poly-Phen-2], Protein Variation Effect Analyzer [PROVEAN], and UniProt alignment) (Table S1). In total, the numbers of chromosomal mutations observed per transconjugant varied from 0 to 2 for the transconjugants harboring IncF plasmids and from 0 to 10 for the transconjugants harboring IncC plasmids (Fig. 2).

The only rearrangement common among all the TR59-IncF and 4/16 TR46-IncC-IncR transconjugants was the IS*150* deletion interrupting the *fliM* gene. FliM is one of three proteins (FliG, FliN, and FliM) that form the rotor-mounted switch complex, located at the base of the basal body. This complex acts on the direction of flagellar rotation (48). E. coli J53-2 is a nonmotile strain because the gene encoding FliM is interrupted by an IS*150* insertion. By observing growth on semisolid agar plates, we confirmed that motility was restored for all the TR59-IncF and TR46-IncC-IncR transconjugants that had lost the IS*150* element (data not shown).

**FIG 3:**
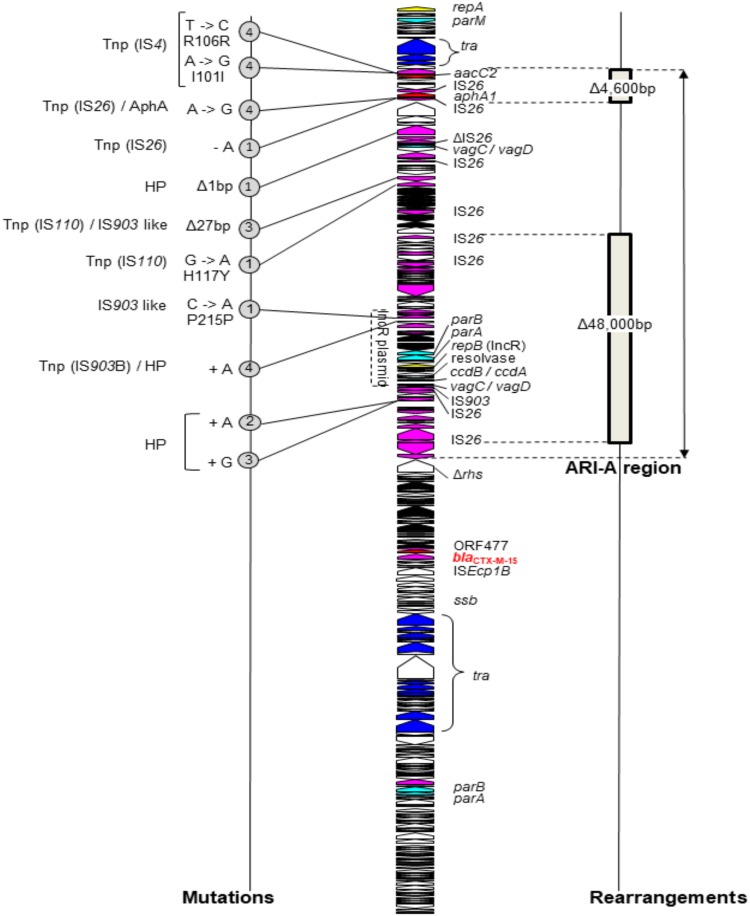
Mutations (left) and rearrangements (right) detected in the genome of the pRCS46 (IncC-IncR, pST3) plasmid (middle) after conjugation in E. coli J53-2. A linear map of the plasmid is presented, and open reading frames are shown as arrows indicating the direction of transcription (dark blue, plasmid transfer; yellow, replication; light blue, plasmid maintenance; red, resistance; black, metabolism; pink, mobile elements; white, hypothetical proteins). The *bla* gene is indicated in red. The IncR plasmid is indicated by a dotted line. The region inserted at the integration spot ARI-A is indicated by a double arrow. For rearrangements, the two deletions correspond to those observed in 11 plasmids. For mutations, the number of mutated plasmids is indicated in a circle, with the mutation and the gene product at the left. Δ, deletion; HP, hypothetical protein; Tnp, transposase; AphA, aminoglycoside 3′-phosphotransferase.

For plasmid DNA, none of the three IncF plasmids showed modifications. Only one intergenic mutation was detected in the IncC plasmid pRCS30 of a single transconjugant. In contrast, many events (mutations and deletions) were detected in the IncC-IncR plasmid pRCS46, all in a large region of 93,032 bp inserted at the integration spot ARI-A (45) (Fig. 3). Eleven mutations were detected in seven TR46 transconjugants, eight of them in genes encoding transposases. Two deletions were observed in 11 TR46 transconjugants: an IS*26*-mediated deletion of a 48,000-bp sequence carrying a complete backbone of an IncR plasmid and an IS*26*-mediated deletion of a 4,600-bp sequence carrying resistance modules (an *aacC2* module and an IS*26-aphA1* unit) (49, 50). The sensitivity to aminoglycosides (kanamycin, gentamicin, tobramycin, and amikacin) of the latter transconjugants was restored, as shown by MIC measurements (Table S2).

### Fitness measurements of evolved TR59-IncF and TR46-IncC-IncR clones.

To evaluate the adaptation of CTX-M-15-encoding plasmids in E. coli, we conducted an evolution experiment with an E. coli J53-2 plasmid-free strain, a TR59-IncF transconjugant, and a TR46-IncC-IncR transconjugant harboring a non-IS*26*-deleted pRCS46 plasmid. Five replicates of each strain at day 0 of the evolution assay (J53-2^0^, TR59^0^, and TR46^0^) were evolved in parallel in lysogeny broth (LB) by daily serial transfer (Fig. S3). Concomitantly, five additional replicates of TR59 and TR46 (TR59^0^-C and TR46^0^-C) were evolved in LB containing 2 mg/liter of cefotaxime. After 112 days (1,120 generations), we isolated five independent clones for each of the evolved strains (lineages) (J53-2^112^, TR59^112^, TR59^112^-C, TR46^112^, and TR46^112^-C) (Fig. S3). For J53-2^112^-5, TR46^112^-5, and TR46^112^-C3 clones, further sequencing analyses did not allow us to exclude intralineage contamination. Therefore, we chose to exclude them from the rest of our study. Ultimately, we measured the MGRs of five independent evolved clones from the TR59^112^ and TR59^112^-C lineages and four independent evolved clones from the J53-2^112^, TR46^112^, and TR46^112^-C lineages. As no significant fitness cost variation was detected between the MGRs of the evolved clones belonging to the same lineage, further statistical analyses were performed after pooling the MGRs of the clones from each lineage. The MGRs of J53-2^112^ clones were the highest (2.18 div h^−1^) (Fig. 1B). The differences between the MGRs of J53-2^112^ clones and the MGRs of TR59^112^ (2.12 div h^−1^) or TR59^112^-C (2.14 div h^−1^) clones were nonsignificant, while the difference between the MGRs of J53-2^112^ clones and the MGRs of the TR46^112^ clones (2.07 div h^−1^) or the TR46^112^-C clones (2.08 div h^−1^) were significant (*P* = 0.025 and *P* = 0.002, respectively) (Fig. 1B). The negative impact of the IncF pRCS59 plasmid was compensated for until it became nonsignificant, while the negative impact of the IncC-IncR pRCS46 plasmid was maintained regardless of antibiotic pressure.

After 1,120 generations in the absence of antibiotic pressure, plasmid loss was not observed for any of the transconjugant clones.

### Whole-genome analysis of TR59-IncF and TR46-IncC-IncR evolved clones.

To determine whether mutational events, occurring during *in vitro* evolution, could explain host adaptation to plasmid carriage, we sequenced the evolved clones and compared the events that occurred after 1,120 generations to those observed in the transconjugants immediately after conjugation (ancestral transconjugants). All events described above in ancestral transconjugants were found in the evolved clones, showing that DNA modifications detected after conjugation were stable during evolution.

The additional modifications identified were only in chromosomal DNA: six in J53-2 clones (three intergenic and two nonsynonymous point mutations and one IS*5* insertion), five in TR59-IncF clones (one intergenic, three nonsynonymous, and one synonymous point mutations), and six in TR46-IncC-IncR clones (five nonsynonymous point mutations and one prophage deletion) (Fig. S4 and Table S3). Among the nonsynonymous point mutations, six were predicted to be deleterious (Table S3). Two of these mutations were found in the ribonuclease-encoding genes *rnhB* and *rne* (51, 52), each in one a TR59-IncF clone. Two other mutations, each in one a TR46-IncC-IncR clone, concerned genes directly or indirectly involved in the transcriptional regulation of genes used for a wide variety of biological stress-associated functions: *rpoN*, encoding the alternative sigma 54 factor, and *yaiB* (also named *iraP*), encoding the antiadapter IraP protein, which is known to interfere with RssB-dependent degradation of RpoS and which increases σ stability upon starvation or under certain stress conditions (53). To detect the occurrence of these mutations during evolution, we performed specific PCR targeting mutations followed by Sanger sequencing at four times of evolution (days 24, 47, 75, and 112). We observed that the mutations occurred throughout evolution (Table S3).

### Variations in phenotypic traits of the evolved TR59-IncF and TR46-IncC-IncR transconjugant strains.

We examined whether the evolved transconjugants (TR59^112^, TR59^112^-C, TR46^112^, and TR46^112^-C) underwent changes in phenotype traits such as motility, antibiotic resistance, and plasmid transfer rate. All the evolved clones were mobile like the ancestral transconjugants. Antibiotic susceptibilities of the ancestral and evolved transconjugants, determined by disk diffusion assays, showed no significant differences. More precisely, the MICs of cefotaxime and ceftazidime and of other antibiotics to which the transconjugants were resistant (tetracycline, tobramycin, and gentamicin for TR59^112^ and TR59^112^-C and kanamycin, gentamicin, and amikacin for TR46^112^ and TR46^112^-C) showed no significant differences.

We compared, by conjugation into E. coli DH10B, the transfer rates of plasmids pRCS59 (IncF) and pRCS46 (IncC-IncR) before and after evolution, using a method described previously by Simonsen et al. (54). The transfer rate of plasmid pRCS59 was very low, below our detection level (estimated at 10^−12^), and lower than the transfer rate of plasmid pRCS46, detected at between 10^−11^ and 10^−12^. No significant difference was observed between transfer rates of ancestral and evolved pRCS46 plasmids, regardless of the presence or absence of cefotaxime during evolution.

## DISCUSSION

Although large conjugative plasmids impose a fitness cost on the bacterial host in the absence of selective pressure (25), they play a key role in the dissemination of CTX-M (16, 20). Current knowledge of CTX-M enzyme dissemination shows that the CTX-M-15 and CTX-M-14 variants are the most prevalent in all regions of the world (4). Some epidemic plasmids, associated with certain high-risk clones, contribute to the explosive dissemination of *bla*_CTX-M_ genes (10). The spread of *bla*_CTX-M-15_ in human E. coli isolates is globally associated with IncF plasmids, linked to the spread of an E. coli ST131 O25b:H4 *H*30Rx/C2 sublineage (4, 13, 14). Here, we investigated, in a J53-2 E. coli strain, the cost of plasmids of two Inc groups, IncF (including RST F2:A1:B-plasmids) and IncC plasmids. In each group, the plasmids carried a *bla*_CTX-M-15_ or *bla*_CTX-M-14_ gene.

As previously described (22, 24, 55), all tested plasmids imposed a significant burden on the recipient strain immediately after conjugation, regardless of the plasmid Inc group, the type of multiresistance region (MRR) that they carried, or the type of CTX-M produced.

During experimental evolution, we found that the initial fitness cost of carriage of two of the *bla*_CTX-M-15_-containing plasmids, the F2:A1:B- IncF pRCS59 plasmid and a non-IS*26*-deleted IncC-IncR pRCS46 plasmid, in E. coli strain J53-2 (TR59-IncF and TR46-IncC-IncR, respectively), was compensated for in the IncF plasmid but not in the IncC-IncR plasmid, regardless of the presence or absence of antibiotic pressure.

To understand the difference in fitness cost compensation between the two plasmids, we examined traits that have been described to counteract fitness cost. We observed no phenotypic silencing of antibiotic resistance genes in the absence of antibiotics that could confer fitness advantages (56). No loss of plasmid was observed during evolution for TR59-IncF or TR46-IncC-IncR. This was not surprising, as besides partition systems, the plasmids carry several addiction systems (four on the pRCS59 IncF plasmid and three on the pRCS46 IncC-IncR plasmid) that prevent the appearance of plasmid-free segregants under nonselective conditions (57–59). Thus, the alleviation of the fitness cost of IncF CTX-M-15 plasmid pRCS59 carriage after evolution without antibiotic pressure was not caused by the emergence of plasmid-free cells in the population. The persistence of a plasmid in bacterial populations can be accomplished by a high transfer rate, which can compensate for any fitness cost associated with its carriage (25, 59, 60). As described previously for large conjugative multidrug-resistant plasmids (22, 29, 60), the *in vitro* conjugation efficiency was low for the IncC-IncR pRCS46 plasmid and even lower for the IncF pRCS59 plasmid, with no detectable difference between ancestral and evolved plasmids.

To understand the burden of IncF and IncC-IncR plasmids on E. coli during conjugation, we performed WGS of all transconjugants immediately after the transfer of plasmids. Chromosomal and plasmid modifications mostly occurred in transconjugants carrying the CTX-M-15 IncC-IncR plasmid pRCS46. The absence of mutation in the genes encoding DNA repair enzymes in this ultimately highly mutated transconjugant allowed us to exclude a hypermutator phenotype (61). Thus, the impact on the bacterial host chromosome is putatively plasmid, but not Inc group, dependent. Sequencing of the plasmids showed that 68% of the CTX-M-15 IncC-IncR pRCS46 plasmids had IS*26*-mediated deletions, whereas no rearrangement was detected in the IncF plasmids in spite of many IS*26* insertions being present. IS*26* has been shown to play a major role in the reorganization of multiresistant plasmids (62, 63). An IS*26*-mediated deletion alleviating the fitness cost of plasmid carriage was described previously (50). In the non-IS*26*-deleted CTX-M-15 IncC-IncR pRCS46 plasmids, the mutations observed were not in the plasmid backbone but in the large region inserted at the ARI-A integration spot, mainly in transposases, indicating that this region is likely costly.

As previous studies have highlighted that compensatory mutations occurring during evolution can reduce the cost of plasmid carriage for daughter cells (32–34), we compared by WGS the genomes of the evolved transconjugants to those of the ancestral transconjugants. In contrast to the many modifications seen immediately after conjugation, no additional mutation or rearrangement and no loss of a costly region were seen in either evolved plasmid. Thus, once in the host strain, the non-IS*26*-deleted IncC-IncR plasmid pRCS46 is stably maintained without undergoing modification, as is the IncF plasmid pRCS59. Since the IncC-IncR pRCS46 plasmid is 40 to 50% larger than the other plasmids tested, the cost of this plasmid could simply be due to the energetic burden. However, the absence of an IS*26*-mediated deletion in the plasmid throughout the evolution experiment is against this hypothesis. The numbers of genomic modifications detected over the course of evolution, appearing evenly over time, are similar for the transconjugants and the control strain J53-2. This suggests that mutations occurring during evolution were not under strong selection and are unrelated to the presence of the plasmid. However, mutations predicted to be deleterious were more frequently observed for TR46-IncC-IncR than for TR59-IncF or J53-2 clones. There were too few mutations, never found within the same operon or locus, to detect a clear signal of convergence between lineages.

Our study has several limitations. First, we performed the experiments in a unique laboratory host strain. Indeed, the genetic background of strains plays an important role in bacterium-plasmid adaptation, and evolutionary mechanisms promoting plasmid-host long-term adaptation are not universal (37, 38). Further investigations should be performed in more relevant clinical models, including various E. coli backgrounds allowing comparative adaptations. However, we performed conjugation assays of the pRCS59 plasmid in E. coli ST131 and ST95 strains that were not successful, as these strains carried no curable IncF plasmids (data not shown). Second, changes in chromosomal and/or plasmid gene expression, without changes in coding sequences, have been demonstrated to be sufficient to ameliorate the fitness cost of the carriage of a plasmid in some cases (33, 35, 64). Therefore, a transcriptional analysis should be undertaken that could probe the potential impact of gene expression on the alleviation of the cost of the F2:A1:B- IncF-type CTX-M-15 plasmid in E. coli. Nevertheless, our results showed a clear difference in the evolution of the fitness cost between two plasmids, showing better adaptation of the F2:A1:B- IncF-type CTX-M-15 plasmid than of the IncC-IncR-type CTX-M-15 plasmid.

In conclusion, segregation control exerted by addiction systems maintained both the IncF CTX-M-15 plasmid of RST F2:A1:B- and the IncC-IncR CTX-M-15 plasmid in the E. coli J53-2 host strain. However, the IncF CTX-M-15 plasmid is maintained without a fitness cost, unlike the IncC-IncR CTX-M-15 plasmid. We hypothesize that in the absence of selective pressure, the E. coli strain carrying the poorly transferrable IncF CTX-M-15 plasmid of RST F2:A1:B- may persist and spread in bacterial populations, whereas the E. coli strain carrying the IncC-IncR CTX-M-15 plasmid is more likely to be outcompeted. Our findings indicate that the IncF CTX-M-15 plasmid is well adapted to the E. coli strain studied, contrary to the IncC-IncR CTX-M-15 plasmid, and that such plasmid-host adaptation could participate in the evolutionary success of the CTX-M-15-producing pandemic E. coli ST131-O25b:H4 lineage.

## MATERIALS AND METHODS

### Bacterial strains and plasmids.

We selected five CTX-M-encoding plasmids from a collection of recently sequenced plasmids (40) on the basis of their host range, their replication control type, and the type of *bla*_CTX-M_ gene that they carried. They included three narrow-host-range IncF plasmids, pRCS59 (GenBank accession number LT985271), pRCS102 (accession number LT985213), and pRCS65 (accession number LT985277), and two broad-host-range IncC plasmids, pRCS30 (accession number LT985224) and pRCS46 (accession number LT985249) (see Fig. S1 in the supplemental material). The plasmids pRCS59 (130,560 bp) and pRCS102 (129,394 bp) have two replicons, FII and FIA, of RST F2:A1:B- and carry similar MRRs containing the same three resistance modules [a *tet*(A) module, an *aacC2* module, and an IS*26*-mediated cassette array (IS*26-aacA4-cr-bla*_OXA-1_-*catB3*Δ-IS*26*)] (22, 40, 65) and a *bla*_CTX-M-15_ transposition unit. These plasmids originated from fluoroquinolone-resistant E. coli strains of phylogroup B2, ST131 according to the Achtman multilocus sequence typing (MLST) scheme (ST43 according to the MLST Pasteur scheme [66] [https://bigsdb.pasteur.fr/ecoli/ecoli.html]), and serotype O25b:H4 (strains CB661 and CB708, respectively), isolated from urinary tract infections in adult patients in 2002 (Paris area, France). Plasmid pRCS65 (121,498 bp) has one FII replicon of RST F2:A-:B- and carries an MRR containing *bla*_TEM-1_, an *aacA4-cmlA* integron, *mphA*, *ermB*, a mercuric operon, and a *bla*_CTX-M-14_ transposition unit. The plasmid originated from an E. coli strain of phylogroup A and ST2 (MLST Pasteur scheme) (strain CB522) isolated from a urinary tract infection in an adult patient in 1995 (Paris area, France). Plasmid pRCS30 (157,836 bp) of pST3 carries on the one hand a *bla*_CTX-M-14_ transposition unit and on the other hand a group of four resistance genes (*sul2*, *strA-strB*, *ermMI*, and *bla*_TEM-1_) inserted at two different integration spots, named ARI-A and ARI-B by Harmer and Hall (44, 45), respectively. This plasmid originated from an E. coli strain of phylogroup F and ST721 (MLST Pasteur scheme) (strain CB513) isolated from a case of bacteremia in an adult patient in 1999 (Paris area, France). Plasmid pRCS46 (216,620 bp) of pST3 carries a *bla*_CTX-M-15_ transposition unit which is inserted downstream of a methylase-encoding gene. On this plasmid, a large region of 93,032 bp is inserted at the integration spot ARI-A (45). This region contains two resistance modules (an *aacC2* module and an IS*26*-mediated *aphA1* unit) (67, 68) and the complete backbone sequence of an IncR plasmid (17,000 bp) (69). The plasmid is thus a composite plasmid, IncC-IncR, that contains two replicons. This plasmid originated from an E. coli strain of phylogroup A and ST718 (MLST Pasteur scheme) (strain CB195) isolated from a case of fecal carriage in an adult patient in 1999 (Paris area, France).

### Conjugation assays.

We performed multiple independent transfer assays by conjugation of the ESBL-encoding plasmids (pRCS59, pRCS102, pRCS65, pRCS30, and pRCS46) from the donors (CB661, CB708, CB522, CB513, and CB195, respectively) to E. coli J53-2 as the recipient strain (K-12 F Δ*pro* Δ*met* Rif^r^) (70). From a glycerol stock, the donor strains and the J53-2 recipient strain were plated on LB agar and grown overnight at 37°C. At this step, 21 randomly selected colonies of J53-2 (named J53-2^rec^) were maintained for use in further evolution experiments, fitness assays, and genomic analyses. For independent conjugation assays, we used 10 to 20 randomly selected colonies of each donor strain and of the J53-2 recipient strain. Donors and recipients were grown overnight in 5 ml of LB at 37°C under constant stirring at 200 rpm. Next, 100 μl of the culture was transferred into 10 ml of fresh LB and incubated for 2 h under the same conditions to obtain an exponential-phase population. For mating experiments, 2 ml of culture of the donor strain and 2 ml of culture of the J53-2 recipient strain were mixed and incubated at 37°C without shaking for 50 min. Concomitantly for each conjugation assay, 2 ml of the culture of the recipient strain J53-2 was incubated under the same conditions. The mating cultures were plated on LB agar plates containing rifampin (250 mg/liter) and cefotaxime (2.5 mg/liter) for transconjugant selection, and the J53-2 incubation control cultures (named J53-2^inc^) were plated on LB agar. Finally, we obtained 6 transconjugants (TRs) for the plasmid pRCS59 (TR59-1 to TR59-6), 4 for the plasmid pRCS102 (TR102-1 to TR102-4), 4 for the plasmid pRCS65 (TR65-1 to TR65-4), 10 for the plasmid pRCS30 (TR30-1 to TR30-10), and 16 for the plasmid pRCS46 (TR46-1 to TR46-16).

The transconjugants and 20 of the J53-2^inc^ strains were stored at −80°C in LB containing 30% glycerol for further experiments.

### Evolution experiments.

The strains were inoculated into 5 ml of LB and grown under constant shaking at 200 rpm. After overnight incubation at 37°C, 5 μl of the culture was inoculated into five tubes containing 5 ml of fresh LB. For the transconjugants TR59-IncF and TR46-IncC-IncR, five additional tubes containing LB plus cefotaxime (2 mg/liter) were inoculated. At this step, the 25 cultures were considered the ancestors of each lineage (day 0 of the evolution assay; denoted X^0^). Next, the 25 cultures were propagated by daily serial transfer, with a 1/1,000 dilution (5 μl of culture grown overnight diluted into 5 ml of fresh LB with and without cefotaxime), for 112 days (corresponding to 10 generations per day; 1,120 generations in total) and incubated overnight at 37°C under constant stirring at 200 rpm. Finally, after 112 days, 25 independent lineages were obtained (denoted X^112^ and X^112^-C for the lineages under cefotaxime pressure) (Fig. S3). At day 0 and every 5 or 6 days of the evolution assay, a culture sample from each lineage was collected and stored at −80°C with glycerol for future analysis. Prior to each storage step, the culture was isolated on LB agar for purity, and antibiotic susceptibility was tested by disk diffusion. At days 58 and 112, the presence of the *bla*_CTX-M-15_ gene in the clones was confirmed by PCR using primers targeting IS*Ecp1* and open reading frame 477 (ORF477) (Table S4).

### Individual fitness assays.

Fitness, defined here as the MGR, was determined from growth curves using a high-precision technique developed in-house (see the supplemental material) (71). The optical density was monitored using a homemade turbidimeter, which allows quasicontinuous, parallel turbidimetric measurement of cultures growing in 15-ml glass culture tubes (5 ml of culture grown overnight in 5 ml of LB) at 37°C in a standard shaking incubator at 200 rpm. Each tube has its own light source (∼700-nm-peak-wavelength LED) and phototransistor, and measurements are recorded every 10 s for a range of emitted light intensities. Different light intensities provide optimal results for different cell density ranges, and so appropriate intensities can be selected after data collection depending on the growth phase of interest (lower intensity for lower cell density and vice versa). For each strain, the fitness assay was repeated six times. The measurements were collected using a Python script developed in our laboratory, and the MGR was obtained by the lm function in R (http://www.R-project.org/).

### Whole-genome sequencing.

WGS was performed using Illumina technology. Briefly, DNA was prepared for indexed, paired-end sequencing on the Illumina HiSeq 2500 system (Integragen, MA, USA) for the evolved strains and on the Illumina MiSeq system (Integragen, MA, USA) for all other strains. DNA samples were extracted using the genomic DNA NucleoMag tissue kit from Macherey-Nagel, the sample concentrations were normalized at 2 nmol/liter, and tagmentation was processed using the Nextera DNA library preparation kit. The pooled paired-end libraries were sequenced to a read length of 2 by 100 bp with Illumina HiSeq 2500 reagent kit V4 and to a read length of 2 by 300 bp with Illumina MiSeq reagent kit V3. The genomes were sequenced at an average depth of 50×.

In addition, using single-molecule real-time sequencing by Pacific Biosciences RS II technology, we sequenced DNA from the TR59° and TR46° ancestral strains used for evolution experiments. Library preparation and sequencing were performed by GATC Biotech (Constance, Germany).

### DNA sequence analysis and comparison.

As the E. coli J53-2 strain used in this study is a K-12 derivative, we used the E. coli K-12 MG1655 strain, available on the MaGe platform (47), as a reference chromosomal genome, whereas we used the plasmid sequences as the reference for comparison of plasmid genome evolution. To identify point mutations and rearrangements, we compared sequence reads using Breseq 0.27.1 (46) and the PALOMA evolution platform from the MicroScope website (https://www.genoscope.cns.fr/agc/microscope/mage/viewer.php).

Mutations or rearrangements common to all J53-2 genomes, which are a large IS*5*-mediated *proBA* operon deletion and mutations of *metF* (GAG→AAG; E28K), *rpoS* (CAG→TAG; Q33*), and *rpoB* (CAC→TAC; H526Y), were excluded from the comparative analysis. The deletion and the two first mutations corresponded to the J53 strain genotype (70), whereas the fourth corresponded to the rifampin resistance phenotype of the J53-2 strain, confirming the validity of our sequencing methods and data analysis.

Nonsynonymous gene mutations were analyzed with three distinct software tools to predict the possible impact of an amino acid substitution on the function of the bacterial protein: SIFT 4G (http://sift.bii.a-star.edu.sg/sift4g), PROVEAN (http://provean.jcvi.org/index.php), and Poly-Phen 2 (http://genetics.bwh.harvard.edu/pph2). We also looked for the frequency of each nonsynonymous mutation in the sequences available in the UniProt database (http://www.uniprot.org/). We concluded that a substitution was deleterious when at least two of the three software packages predicted damage to the function of the bacterial protein, with less than 10% of the mutated gene found in the UniProt database (72).

To confirm point mutations found with the pipeline analysis, and to date their occurrence during evolution experiments, specific PCRs framing the mutations (Table S4) followed by Sanger sequencing (Genewiz, Beckman Coulter Genomics, UK) were performed on the evolved clones at four different times of evolution (days 24, 47, 75, and 112).

### Antimicrobial susceptibility testing.

Antimicrobial susceptibility testing by the disk diffusion method (ampicillin, ticarcillin, piperacillin, amoxicillin-clavulanic acid, ticarcillin-clavulanic acid, piperacillin-tazobactam, cefotaxime, ceftazidime, aztreonam, cefoxitin, cefepime, ertapenem and imipenem, sulfonamides, trimethoprim, doxycycline and tetracyclines, aminoglycosides [kanamycin, tobramycin, streptomycin, gentamicin, amikacin, and netilmicin], nalidixic acid, ofloxacin and ciprofloxacin, rifampin, fosfomycin, and chloramphenicol) and determination of the MICs of cefotaxime, ceftazidime, kanamycin, gentamicin, tobramycin, and amikacin, using the Etest method, were performed according to European Committee on Antimicrobial Susceptibility Testing (EUCAST) guidelines (http://www.eucast.org).

### Plasmid stability.

Spontaneous plasmid loss quantification was performed on the plasmid-carrying strains evolved without antibiotic pressure at 750 and 1,120 generations. From the frozen culture, 10-fold-diluted samples (10^−2^ to 10^−7^) were spread onto LB agar plates to obtain individual colonies. From the plates, 188 colonies were each suspended in a single well of 96-well microtiter plates (1 colony per well) in LB. In the two remaining wells of each plate, control isolates were suspended (J53-2 and TR59-IncF or TR46-IncC-IncR). Agar plates containing 2 mg/liter of cefotaxime for TR59 and TR46 and 20 mg/liter of kanamycin for TR46 were spotted with 2 ml of each well. After overnight incubation at 37°C, every spot was marked as “growth” or “no growth,” indicating the presence or absence of the plasmid, respectively. Plasmid stability was calculated as the ratio of resistant cells to total cells.

### Transfer rate measurements.

To estimate the rate of transfer, conjugal transfer of plasmids was performed using the method of Simonsen et al. (54), which is based on a single endpoint estimate of the donor, recipient, and transconjugant densities and the population growth rate. Transfer of the ancestral and evolved plasmids at 112 days was accomplished using the E. coli DH10B strain as the recipient, which is streptomycin resistant. Briefly, donors and recipients were grown overnight in 10 ml of LB without antibiotic selection. The next day, bacterial cultures were diluted and mixed at a ratio of 1:1 (by volume) and incubated at 37°C for pRCS59 and at 30°C for pRCS46, under constant stirring at 50 rpm for 6 h (time to reach stationary phase). The mixed broth culture was spread at 0 h and 6 h onto 3 different LB agar plates containing cefotaxime (2 mg/liter), streptomycin (30 mg/liter), and both. Final densities of donors (*D*), recipients (*R*), and transconjugants (*T*) were determined by counting colonies on agar plates. The measurement of the optical density of the mixed culture was performed at two times, 3 h and 6 h, at 670 nm. The growth rate per hour (ψ) of mating cultures was estimating by regressing the natural log (ln) of the total density (*N* = *T* + *R* + *D*) versus time during exponential phase. We estimated the conjugation rate (γ) using the formula of Simonsen et al. (54), γ = ψ × ln(1 + (*T*/*R*)(*N*/*D*)]/(*N* – *N*_0_), where *N*_0_ is the initial population size. This measure is in units of millimeters per cell per hour, indicating the volume, during 1 h, in which a plasmid-bearing cell can successfully “search for” and infect a plasmid-free cell. The presence of the *bla*_CTX-M-15_ gene in the transconjugants was confirmed by antibiotic susceptibility and PCR. Three replicates were performed for each strain combination.

### Motility assay.

Ancestral and evolved strains were grown overnight in LB medium, washed, and suspended in isotonic water to a concentration of 1 × 10^2^ cells ml^−1^. About 10 cells were spotted onto semisolid LB agar dishes containing 0.35% agar. Swim plates were incubated for 48 h at 37°C in a humid atmosphere. Experiments were repeated 2 times. For the data analysis, only two categories were considered (motile and nonmotile) (73). The nonmotile category was defined by growth limited to the initial spot size, and the motile category was defined by growth of more than 1 cm from the initial spot size.

### Statistics.

For statistical analyses of data from conjugation assays, we carried out unpaired Student’s test, and *P* values of <0.05 were considered significant. For statistical analyses of data from the fitness assays, we used analysis of variance (ANOVA) and unpaired Student’s test using R software, with *P* values of <0.05 being considered significant.

### Accession number(s).

All reads have been deposited at the European Nucleotide Archive (project accession number PRJEB32706).

## Supplementary Material

Supplemental file 1
