# Supplementary material for: Advantage of the F2:A1:B- IncF Pandemic Plasmid over IncC Plasmids in In Vitro Acquisition and Evolution of blaCTX-M Gene-Bearing Plasmids in Escherichia coli
Source: Antimicrob Agents Chemother. 2019 Sep 23;63(10):e01130-19. doi: 10.1128/AAC.01130-19 (PMC6761558; doi:10.1128/AAC.01130-19)
Supplement: Supplemental file 1 [file AAC.01130-19-s0001.pdf]

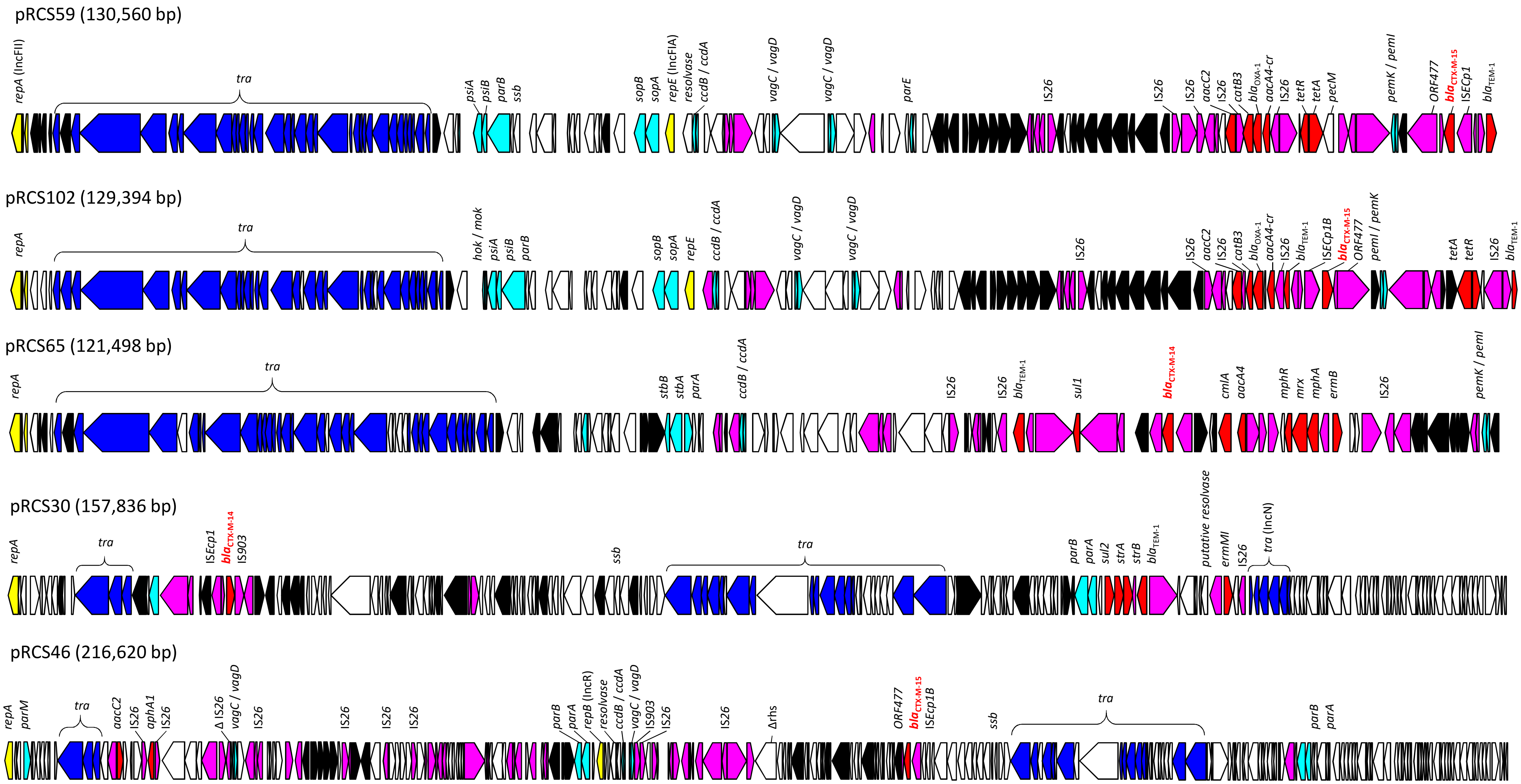

FIG S1: Linear maps of pRCS59 and pRCS102 (IncF, F2:A1:B-), pRCS65 (IncF, F2:A-:B-), pRCS30 (IncC, pST3) and pRCS46 (IncC-IncR, pST3) plasmids. Open reading frames are shown as arrows indicating the direction of transcription. Dark blue, plasmid transfer; yellow, replication; light blue, plasmid maintenance; red, resistance; black, metabolism; pink, mobile elements and white, hypothetical proteins. *bla* genes are noted in red.

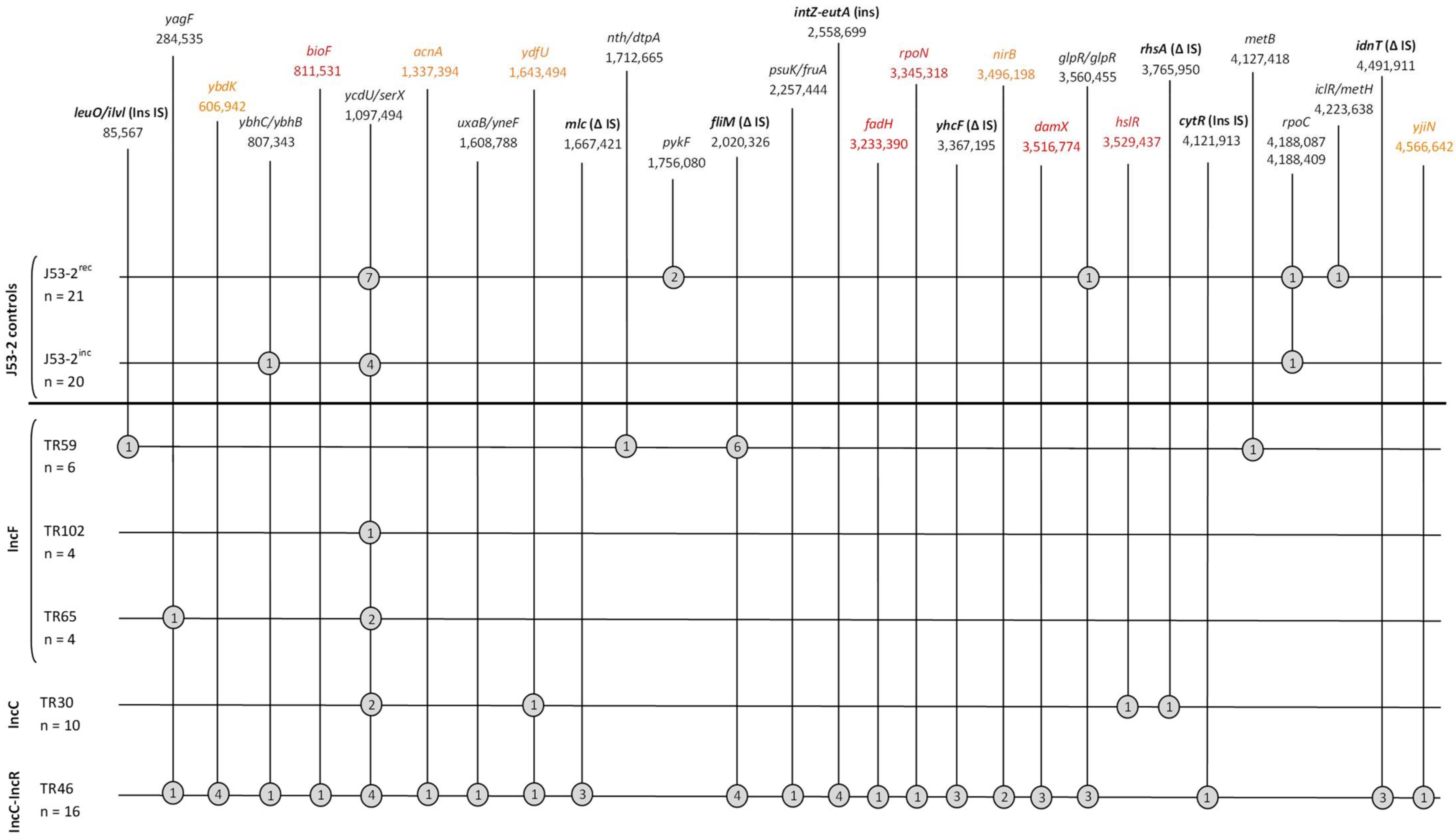

FIG S2: Mutations and rearrangements detected after conjugation in the chromosome of each lineage compared to reference *E. coli* K-12 MG1655 genome. For each lineage, n is the total number of strains, the numbers of mutated strains are indicated in the circles. J53-2rec and J53-2inc corresponds to strains J53-2, respectively, before and after an exponential growth phase (see Materials and Methods). The genes are ordered from the thr locus, with below the distance in bp (site of mutation). For rpoC, two sites are mutated. The intergenic and synonymous mutations are in black, non-synonymous mutations interpreted as deleterious are in dark red and non-deleterious in light red, and rearrangements are in bold (see Table S1 for more details on the mutations and rearrangements). Ins, insertion; Δ, deletion.

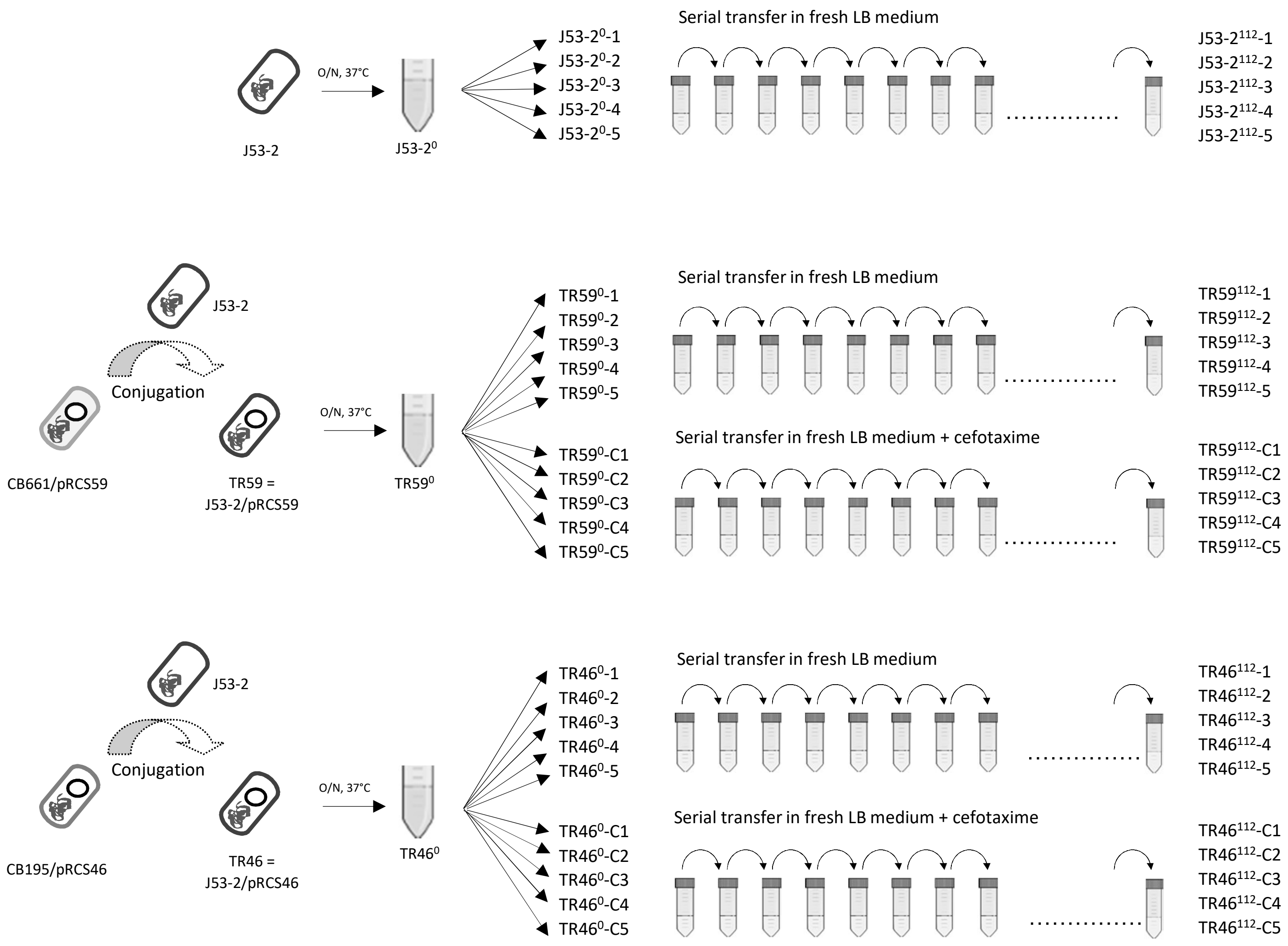

FIG S3: Design of the experimental evolution assay. Three *E. coli* strains were evolved in this study: the transconjugants TR59 and TR46, obtained after transfer of the ESBL plasmids pRCS59 (donor strain: CB66) and pRCS46 (donor strain: CB195) in J53-2 respectively, and the recipient strain J53-2. Using an overnight culture of one randomly selected clone, each strain was inoculated in five tubes containing lysogeny broth (LB) medium and five additional tubes containing LB medium + cefotaxime (2 mg/l) for the transconjugants TR59 and TR46. At this step, the replicates were considered as the ancestral lineages (day 0 of the evolution assay) and were denoted X^0 and X^0-C for the lineages in presence of cefotaxime. After daily serial transfer for 112 days (10 generations per day, 1,120 generations total) of the replicates' cultures, we obtained 25 independent lineages denoted X^{112} or X^{112}-C for the lineages obtained with cefotaxime in the culture.

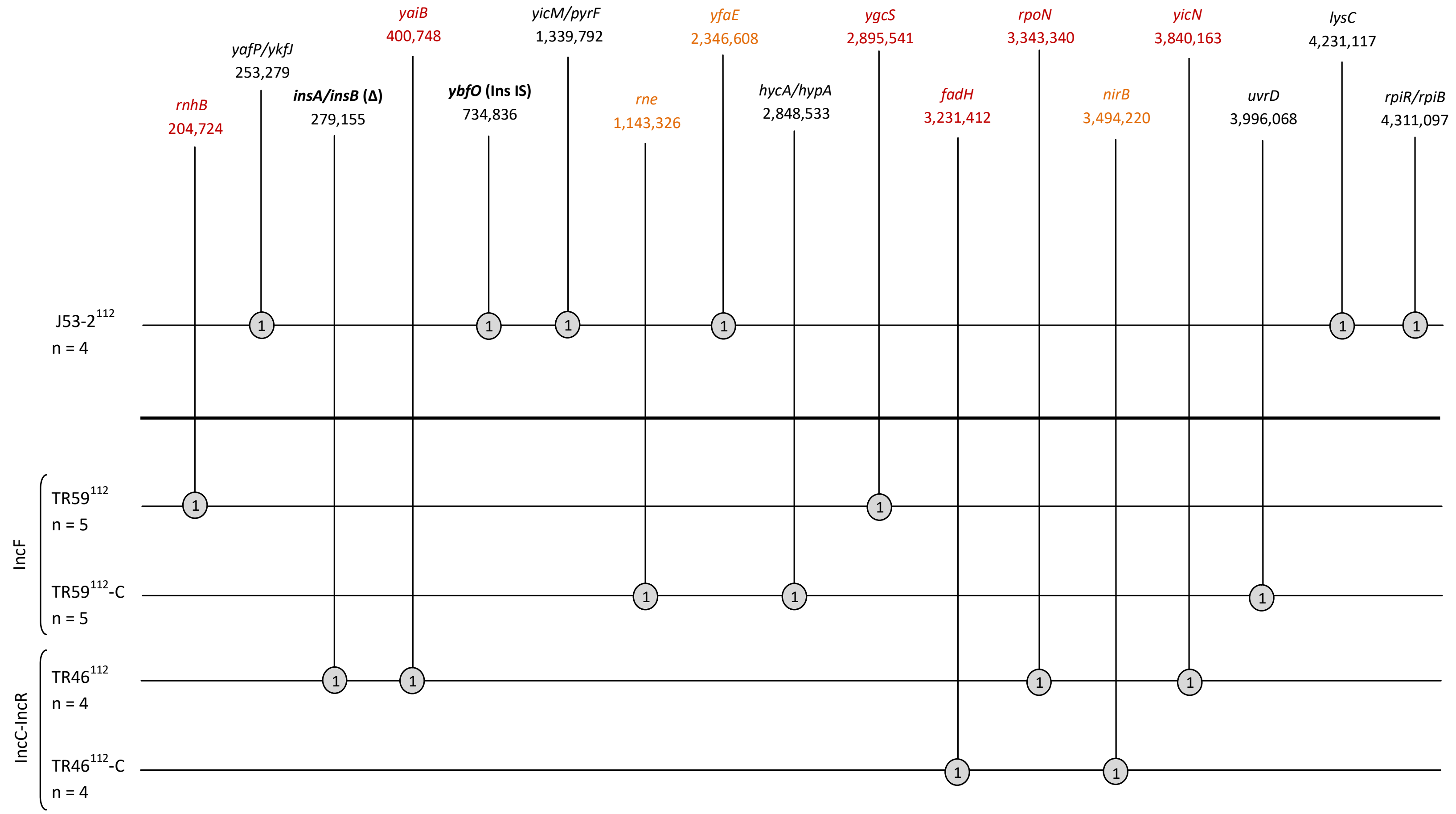

FIG S4: Mutations and rearrangements detected in the chromosome of each evolved lineage at day 112 (day indicated in exponent) with or without cefotaxime (-C) compared to reference *E. coli* K-12 MG1655 genome. For each lineage, n is the total number of strains, the numbers of mutated strains are indicated in the circle. The genes are ordered from the thr locus, with below the distance in bp (site of mutation). The intergenic and synonymous mutations are in black, non-synonymous mutations interpreted as deleterious are in dark red and non-deleterious in light red and rearrangements are in bold (see Table S3 for more details on the mutations and rearrangements). Ins, insertion; Δ, deletion.

TABLE S1: Characterization of mutations and rearrangements in the chromosome of TR59, TR102, TR65, TR30 and TR46 clones.

Gene[a]	Product of gene[b]	Mutation/ Rearrangement[c]	Lineage[d]					Impact of non-synonymous point mutations in coding regions				
			IncF			IncC	IncC-IncR	Prediction score[e]			UNIPROT alignment[f]	Interpretation[g]
			TR59	TR102	TR65	TR30	TR46	SIFT	Poly-Phen	PROVEAN		
leuO/ilvI	Global transcription factor / acetolactate synthase 3 large subunit	Ins IS*5*	1									
yagF	CP4-6 prophage; dehydratase family protein	(GC**G**-> GC**T**) A445A			1		1					
ybdK	Putative gamma-glutamyl cysteine ligase	(**G**TT->**A**TT) V148I					4	0.43	0.064	-0.749	100 %	Non deleterious
bioF	8-amino-7-oxononanoate synthase	(G**A**T->G**G**T) D384G					1	0	0.944	0.601	2.8 %	Deleterious
acnA	Aconitase A	(**G**GC->**A**GC) G522S					1	0.52	0.092	-1.581	0.4 %	Non deleterious
uxaB/yneF	Altronate oxidoreductase, NAD-dependent/putative membrane-associated diguanylate cyclase	(**T**-**G**) -84;+143					1					
ydfU	Qin prophage; uncharacterized protein	(C**A**G->C**T**G) Q209L				1	1	ND	0.017	0.123	32 %	Non deleterious
mlc	Glucosamine anaerobic growth regulon transcriptional repressor; autorepressor	Δ IS*2*					3					
nth/dtpA	DNA glycosylase and apyrimidinic lyase / dipeptide and tripeptide permease A	(**T**-**A**) +507;-104	1									
fliM	Flagellar motor switching and energizing component	Δ IS*150*	6				4					
psuK/fruA	Pseudouridine kinase/fused fructose-specific PTS enzymes: IIBcomponent/IIC components	(**C**-**A**) -126;+297					1					
intZ-eutA	Putative integrase/ethanolamine ammonia-lyase	Ins CPZ55 prophage 6,790 bp					4					
fadH	2,4-dienoyl-CoA reductase	(**G**CC->**A**CC) A576T					1	0	0.982	-4.528	0 %	Deleterious
rpoN	σ54 factor	(**A**GC->**A**TC) S201I					1	0	0.418	-5.377	0 %	Deleterious
yhcF	Putative transcriptional regulator	Δ IS*2*					3					
nirB	Nitrite reductase	(**C**TC->**A**TC) L730I					2	0.1	0	-0.943	4 %	Non deleterious
damX	Cell division protein that binds to the septal ring	(T**T**T->T**C**T) F178S					3	0.02	0.999	-4.764	0 %	Deleterious
hslR	Ribosome-associated heat shock protein Hsp15	(AT**G**->AT**T**) M30I				1		0.41	0.923	-3.945	0 %	Deleterious
rhsA	Rhs protein with putative toxin 55 domain; putative polysaccharide synthesis/export protein; putative neighboring cell growth inhibitor	Δ IS*2*				1						
cytR	DNA-binding transcriptional dual regulator	Ins IS*2*					1					
metB	Cystathionine gamma-synthase	(**C**TA->**T**TA) L242L	1									
idnT	L-idonate and D-gluconate transporter	Δ IS*150*					3					
yjiN	Zinc-type alcohol dehydrogenase like protein	(T**T**G->T**C**G) L202S					1	ND	0	1.868	100 %	Non deleterious

[a]Gene name as in *E. coli* K-12 MG1655.

[b]For intergenic mutations, products of upstream and downstream genes are reported.

[c]Compared to reference *E. coli* J53-2 genome. Nucleotide changes are given in parentheses. The position of intergenic mutations is shown by the relative distance with start or stop codon of genes; Ins, insertion; Δ deletion.

[d]Total number of clones mutated.

[e]SFIT prediction score using SIFT 4G (http://sift.bii.a-star.edu.sg/sift4g), deleterious for SFIT < 0.05; Poly-Phen calcul using Polymorphism-Phenotyping V2 (http://genetics.bwh.harvard.edu/pph2/index.shtml); PROVEAN prediction score using Protein Variation Effect Analyzer (http://provean.jcvi.org), deleterious for score < -2.5. ND, not determined.

[f]frequency detected in the sequences available on the Uniprot database (http://www.uniprot.org/).

[g]Final Interpretation taking into account the four previous metrics (see Materials and Methods).

Table S2: MICs of J53-2, TR59, TR46, and evolved strains TR59 and TR46 at day 112 (day indicated in exponent), with or without cefotaxime (-C).

		MIC (mg/L)						
**Type of plasmid**	**Strain**	CTX	CAZ	K	GM	TM	AN	TE
**/**	J53-2	0,03	0,064	0,5	0,094	0,125	0,5	2
**pRCS59 (IncF)**	TR59-1	32	3	4	3	2	1,5	24
	TR59-2	32	2	4	4	3	2	48
	TR59-3	32	2	6	3	4	2	48
	TR59-4	32	2	6	4	3	2	32
	TR59-5	32	1,5	4	4	3	1,5	32
	TR59-6	32	2	6	4	4	2	32
**pRCS46 (IncC-IncR)**	TR46-1	16	1,5	> 256	6	1,5	0,5	2
	TR46-2	16	1	> 256	6	1,5	0,75	1,5
	TR46-3	16	2	> 256	6	1,5	0,5	2
	TR46-4	16	1	> 256	6	1	0,5	1,5
	TR46-5	16	1,5	> 256	4	1	0,5	1,5
**ΔpRCS46 (IncC)**	TR46-6	16	1	0,25	0,064	0,047	0,19	1,5
	TR46-7	16	1	0,25	0,064	0,047	0,19	1
	TR46-8	16	1	0,25	0,064	0,064	0,19	1
	TR46-9	16	1	0,25	0,064	0,064	0,19	1,5
	TR46-10	8	1	0,25	0,064	0,064	0,25	1
	TR46-11	16	1	0,25	0,064	0,047	0,25	1
	TR46-12	16	1	0,25	0,064	0,064	0,25	1
	TR46-13	16	1	0,25	0,064	0,064	0,19	1
	TR46-14	16	1	0,25	0,064	0,064	0,25	1
	TR46-15	16	1	0,25	0,064	0,064	0,19	1
	TR46-16	8	1	0,25	0,064	0,064	0,19	1
**pRCS59 (IncF)**	$TR59^{112}$-1	32	3	6	3	3	1,5	24
	$TR59^{112}$-2	16	3	4	2	3	1,5	24
	$TR59^{112}$-3	32	3	4	3	3	1,5	24
	$TR59^{112}$-4	16	3	3	3	3	1	24
	$TR59^{112}$-5	32	2	3	2	3	1	24
	$TR59^{112}$-C1	32	2	3	3	2	1,5	32
	$TR59^{112}$-C2	32	3	3	3	2	1,5	32
	$TR59^{112}$-C3	32	3	4	2	3	1,5	32
	$TR59^{112}$-C4	32	2	4	3	2	1,5	32
	$TR59^{112}$-C5	32	2	4	3	3	1,5	24
**pRCS46 (IncC-IncR)**	$TR46^{112}$-1	16	2	> 256	6	1	0,5	2
	$TR46^{112}$-2	16	1,5	> 256	4	1	0,75	2
	$TR46^{112}$-3	16	2	> 256	4	1	0,5	2
	$TR46^{112}$-4	16	1,5	> 256	4	1	0,5	2
	$TR46^{112}$-C1	16	1,5	> 256	4	1	0,5	2
	$TR46^{112}$-C2	16	1,5	> 256	4	1	0,5	2
	$TR46^{112}$-C4	16	1,5	> 256	6	1	0,5	1,5
	$TR46^{112}$-C5	16	1,5	> 256	6	1	0,5	1,5

CTX, cefotaxime ; CAZ, ceftazidime ; K, kanamycin ; GM, gentamicin ; TM, tobramycin ; AN, amikacin ; TE, tetracycline

TABLE S3: Characterization of mutations and rearrangements in the chromosome of J53-2, TR59 and TR46 clones occurring during the evolution assay

Lineages	Clones[a]	Gene[b]	Product of gene[c]	Mutation/ Rearrangement[d]	Time of first detection (day)[e]	Impact of non-synonymous point mutations in coding regions				
						Prediction score[f]		Poly-Phen results[g]	UNIPROT alignment[h]	Interpretation[i]
						SIFT	PROVEAN			
J53-2^{112}	J53-2^{112}-1	yciM/pyrF	TPR-repeats- containing protein/orotidine-5'-phosphate decarboxylase	(**A**->**G**) +41; -153	75	-	-	-	-	-
	J53-2^{112}-2	yafP/ykfJ	Putative acyltransferase/ hypothetical protein	(**A**->**T**) +118;-200	75	-	-	-	-	-
		yfaE	Ferredoxin involved with ribonucleotide reductase diferric-tyrosyl radical cofactor maintenance	(**G**CG->**A**CG) A25T	24	0.64	-0.632	0.623	0 %	Non deleterious
	J53-2^{112}-3	lysC	Aspartokinase III	Δ1 bp (**A**, 140)	47	-	-	-	-	-
		rpiR/rpiB	DNA-binding transcriptional repressor/ribose 5-phosphate isomerase B;allose 6-phosphate isomerase	Δ1 bp -83;-276 (**A**)	112	-	-	-	-	-
	J53-2^{112}-4	ybfO	Rhs-like (pseudogene)	Ins IS5	112	-	-	-	-	-
TR59^{112} IncF	TR59^{112}-1	-	-	-	-	-	-	-	-	-
	TR59^{112}-2	-	-	-	-	-	-	-	-	-
	TR59^{112}-3	ygcS	Putative transporter	(**G**GC->**A**GC) G118S	112	0	-5.989	1	6 %	Deleterious
	TR59^{112}-4	rnhB	Ribonuclease HII	(**A**TC->**G**TC) I78V	112	0	-0.972	0.945	0 %	Deleterious
	TR59^{112}-5	-	-	-	-	-	-	-	-	-
	TR59^{112}-C1	rne	Fused ribonuclease-endo ribonuclease	(**A**AC->**G**AC) N89D	75	0.27	-4.875	0.127	0.5 %	Non deleterious
	TR59^{112}-C2	-	-	-	-	-	-	-	-	-
	TR59^{112}-C3	-	-	-	-	-	-	-	-	-
	TR59^{112}-C4	hycA/hypA	Regulator of the transcriptional regulator FhlA/protein involved in nickel insertion into hydrogenases 3	(**G**->**T**) -76;-136	47	-	-	-	-	-
	TR59^{112}-C5	uvrD	DNA- dependant ATPase I and helicase II	(CC**A**->CC**G**) P21P	24	-	-	-	-	-
TR46^{112} IncC-IncR	TR46^{112}-1	yaiB	Anti-RssB factor, RpoS stabilzer during Pi starvation; anti-adapter protein	(**C**GG->**T**GG) R47W	24	0.03	-2.348	0.928	1 %	Deleterious
		yicN	Hypothetical protein	(G**A**A->G**C**A) E88A	112	0.02	-4.120	0.884	0 %	Deleterious
	TR46^{112}-2	insA/insB	IS1 integrases	Δ CP4-6 prophage	112	-	-	-	-	
	TR46^{112}-3	rpoN	σ54 factor	(A**T**C->A**G**C) I201S	112	0	-5.377	0.418	0 %	Deleterious
	TR46^{112}-4	-	-	-	-	-	-	-	-	-
	TR46^{112}-C1	-	-	-	-	-	-	-	-	-
	TR46^{112}-C2	fadH	2,4-dienoyl-CoA reductase	(**A**CC->**G**CC) T576A	24	0	-4.528	0.982	0 %	Deleterious
		nirB	Nitrite reductase	(**A**TC->**C**TC) I730L	24	0.1	-0.943	0	4 %	Non deleterious
	TR46^{112}-C4	-	-	-	-	-	-	-	-	-
	TR46^{112}-C5	-	-	-	-	-	-	-	-	-

[a]Four, ten and eight independents evolved clones for J53-2^{112}, TR59^{112} and TR46^{112}, respectively.

[b]Gene name as in *E. coli* K-12 MG1655.

[c]For intergenic mutations, products of upstream and downstream genes are reported.

[d]Compared to reference *E. coli* J53-2 genome, nucleotide changes are given in parentheses, the position of intergenic mutations is shown by the relative distance with start or stop codon of genes. Ins for insertion, Δ for deletion.

[e]First time point of experimental evolution tested with point mutation or rearrangement detected.

[f]SFIT prediction score (deleterious if < 0.05) using SIFT 4G (http://sift.bii.a-star.edu.sg/sift4g), PROVEAN prediction score (deleterious if < -2.5) using Protein Variation Effect Analyzer (http://provean.jcvi.org).

[g]Poly-Phen result using Polymorphism-Phenotyping V2 (http://genetics.bwh.harvard.edu/pph2/index.shtml).

[h]Frequency detected in the sequences available on the Uniprot database (http://www.uniprot.org/).

[i]Final interpretation taking into account the four previous metrics (see Materials and Methods).

TABLE S4. Primers used to control or date mutations and rearrangements

Name	DNA sequence (5'-3')	Relative position to start codon (n nucleotides)	Amplicon size (bp)
CTX-M-for	TCCGTACAAGGGAGTGTAT	+1226	1228
CTX-M-rev	AGCTTATGGCCTGGTATGCG		
damX-for	GGATGTTTCTCTGCCACCGA	+507	421
damX-rev	GGATGCCGTCTGTACTGGAG		
fadH-for	GTTGTGGCGGGATTGGTTTT	+ 1504	444
fadH-rev	AGCCGCCAATTAAATGCACC		
fliM-for	AGTGTTAGCGGCGAAAGTGA	+35	469
fliM-rev	TTTCCGAACGCACGTACTCA		
hycA_hypA-for	TCCCCTGAACCAGCGAATTG	+456	402
hycA_hypA-rev	AGACCCCAGTTACGCGTTTT		
insA-insB-for	CGACTTTACCCCTGCACAGT	+39	437
insA-insB-rev	GCGTCATCTCGTTAAACGCC		
lysC-for	GCCAACGTGCGTTTAGTTGT	+91	954
lysC-rev	GCTCACTTCTGACGTGGTGA		
nirB-for	CTCGGCGTGGAACTGGAAAA	+1968	435
nirB-rev	CGGCGTATTGACCGTTTCAG		
rnhB-for	AAAGCTGAGCGAAAAACGCC	+141	217
rnhB-rev	CGCAGGCATCGGTAATTTCG		
rne-for	TGAACGTCACGGTTTCCTCC	+186	268
rne-rev	TAATTCGGTACGGTCGTCGC		
rpiR-for	ACCCCAGCAGCTTTGATACC	+124	728
rpiR-rev	TGCGACTTGACTGGCGTAAT		
rpoN-for	ACGAAGTTGAAGCCGTCCTT	+518	397
rpoN-rev	GAGGCGTAGTGCTGGTTGAT		
uvrD-for	AGGCTTGTTGGATCAGACCG	+206	695
uvrD-rev	GCTCCACCGGATTACCGTAG		
yaiB-for	ACTTCCAGACACTATGAAGTTGTGA	- 70	392
yaiB-rev	CAAAGCGACTATAAGTCTCGGC		
ybfO-for	ATTGACCAGGCCAGTAGTGC	+357	842
ybfO-rev	GTTCGGGAGATGTGCTGTCA		
yfaE-for	GCATCACTGGCACACAACTG	+ 18	104
yfaE-rev	GTAACCTTCGCGACACTGGT		
ygcS-for	ATGTTCTCGGCGTCATTGGT	+98	340
ygcS-rev	CCCAGCAAAATACCGCGATG		
yicM-for	TGCCAAAGAGAGCCTGATGG	+76	279
yicM-rev	AGTTGCGATTTCTCCCCTGG		
yicN-for	GTTTTCGGGTGCTGACATCC	+47	292
yicN-rev	CTGACCTGAGCGTCGGTAAT		

Performance of our method to determine growth curve: The apparatus offers several advantages over turbidometric microplate readers, the current method of choice for high-throughput growth measurements of microorganisms in liquid culture. First, the two major problems of growth in microplates, sample evaporation and low aeration (1), are avoided due to the use of standard laboratory culture volumes. Aeration is further improved as the setup allows OD to be measured during continuous agitation, whereas microplate readers require shaking to be stopped for each reading. The standard laboratory conditions used in our system thus allow for better cell growth than do microplates, resulting in a more stable exponential phase and so enabling true exponential growth rate estimates, rather than the alternative, less robust metric of “maximum growth rate”. Second, the ability of our apparatus to measure OD during continuous agitation allows measurements to be taken at extremely high frequency (“quasi-continuously”), allowing easy noise-filtering when necessary and increasing the confidence of growth parameter estimates. Finally, due to the longer path-length in our set-up and the use of a range of light intensities, it is a more sensitive method and so allows growth to be accurately observed at lower cell densities. This permits growth rates to be estimated from earlier in the exponential phase, helping to avoid the many pitfalls of using turbidity to estimate cell number at high cell density/after long growth times (2). According to our results, using the lowest growth difference considered significant, we could establish our method detection limit (2.1 %).

1. Hermann R, Lehmann M, Büchs J. 2003. Characterization of gas-liquid mass transfer phenomena in microtiter plates. Biotechnol Bioeng 81:178–186.

2. Stevenson K, McVey AF, Clark IBN, Swain PS, Pilizota T. 2016. General calibration of microbial growth in microplate readers. Sci Rep 6:38828.
